# Effects of Ca^2+^ and fulvic acids on atrazine degradation by nano-TiO_2_: Performances and mechanisms

**DOI:** 10.1038/s41598-019-45086-2

**Published:** 2019-06-20

**Authors:** Saiwu Sun, Huijun He, Chunping Yang, Yan Cheng, Yongpan Liu

**Affiliations:** 1 College of Environmental Science and Engineering, Guilin University of Technology and Guangxi Key Laboratory of Theory & Technology for Environmental Pollution Control (Guilin University of Technology), Guilin, Guangxi 541004 China; 2 Guangdong Provincial Key Laboratory of Petrochemical Pollution Processes and Control, School of Environmental Science and Engineering, Guangdong University of Petrochemical Technology, Maoming, Guangdong 525000 China; 3 College of Environmental Science and Engineering, Hunan University and Key Laboratory of Environmental Biology and Pollution Control (Hunan University), Ministry of Education, Changsha, Hunan 410082 China; 4 Hunan Dalu Technology Co., Ltd, 559 Yunxi Road, Yuelu, Changsha, Hunan 410036 China

**Keywords:** Environmental impact, Pollution remediation

## Abstract

In this study, the adsorption and UV photocatalytic degradation of atrazine using nano-TiO_2_ particles were studied systematically, and the colloidal stability of nano-TiO_2_ particles in solution was also investigated to reveal the removal mechanism. Experiments which contained the first 6.0 hours darkness and 4.0 hours UV illumination later were conducted at different concentrations of Ca^2+^ and/or fulvic acids (FA) at pH = 7.0. Results showed that the adsorption rate of atrazine onto nano-TiO_2_ particles decreased with the increase of Ca^2+^ and/or FA concentrations, which could be explained well by the colloidal stability of nanoparticles. When the solution contained Ca^2+^ or Ca^2+^-FA, the nanoparticles were aggregated together leading to the decrease of the contact surface area. Besides, there existed competitive adsorption between FA and atrazine on the particle surface. During photocatalytic degradation, the increase of Ca^2+^ and/or FA concentration accelerated the aggregation of nano-TiO_2_ particles and that reduced the degradation efficiency of atrazine. The particle sizes by SEM were in accordance with the aggregation degree of nanoparticles in the solutions. Sedimentation experiments of nano-TiO_2_ particles displayed that the fastest sedimentation was happened in the CaCl_2_ and FA coexistent system and followed by CaCl_2_ alone, and the results well demonstrated the photodegradation efficiency trends of atrazine by nano-TiO_2_ particles under the different sedimentation conditions.

## Introduction

Pesticides are still produced in large quantities and used in agricultural pest and weed control. Most pesticides, such as triazophos and amitrole, are refractory organics and pose a potential threat to ecosystems and man where they are applied^1,2^. As an example, atrazine (2-chloro-4-ethylamino-6-isopropylamino-s-triazine) is one pesticide that has been prevalently used in weed control^3^. Due to its widespread use, atrazine has been detected in surface water and groundwater, which may result in pathological damage such as sexual abnormalities, cancer, thyroid lesions and endocrine disruption^4–6^. Therefore, it is important to minimize the content of atrazine in our living environment, and the maximum concentration of atrazine for drinking water is 0.1 μg/L stipulated by the European Union^7^. Current methods for removing atrazine include physical, chemical, biological and hybrid treatment techniques^8–10^. Among these methods, photocatalysis is an efficient technology that has been extensively studied for the removal of pesticides^11,12^.

During the last two decades, many photocatalytic materials have been produced and used to degrade various pesticides into nontoxic compounds^13–16^. As shown in Table 1, atrazine can be removel by many materials. Among these materials, TiO_2_ and its doped complexes have been widely applied for the elimination of toxic and hazardous organic pollutants^17^. Because of the low toxicity and chemically inert to microorganisms, TiO_2_ is highly efficient in pollutant removal at lower cost^18,19^. Under UV irradiation, TiO_2_ generate electron hole pairs producing reactive species from water (such as hydroxyl radicals) so that pesticides can be degraded^20^.

**Table 1:** Various materials used for atrazine degradation.

Materials	Brief summary	Mechanism	References
Bi_2_O_3_ nanoparticles	UV irradiation, 75% degradation in 1.0 h at pH 6.0	Photocatalysis	Sudrajat and Sujaridworakun^46^
C-Fe_3_O_4_	94% degradation in 1.0 h when C/Fe was 5/1	Adsorption oxidization	Castro *et al*.^47^
N-TiO_2_/ZnS	UV irradiation, 94% degradation in 1.5 h at pH 5.8	Photocatalysis	Sacco *et al*.^48^
graphene oxide-TiO_2_	UV irradiation, 100% degradation in 5.0 h	Photocatalysisadsorption	Cruz *et al*.^24^
Au-Eu_2_O_3_ nanoparticles	visible light irradiation, 100% degradation in 40 min	Photocatalysisadsorption	Aazam^49^
W-TiO_2_	UV irradiation, 100% degradation in 4.0 h	Photocatalysisadsorption	Belver *et al*.^50^
Ag-chitosan	94% degradation	Adsorption	Saifuddin *et al*.^51^
Carbon nanotubes	>90% degradation	Adsorption	Yan *et al*.^52^

Generally speaking, pesticide contaminated wastewater usually contains many different ionic species, such as K^+^, Mg^2+^, NO_3_^−^, Cl^−^ etc., and some natural organic matter^21^. These chemicals can influence the efficiency of photocatalytic materials during contaminants removal. A few studies have reported the effects of chemicals on photocatalytic activity of photocatalytic materials. Wang *et al*. (1999) conducted experiments on the photocatalytic degradation of 2-chloro and 2-nitrophenol by TiO_2_ in aqueous solution. They found that chloride ions seriously inhibited the photocatalytic reaction at pH 3.0, nitrate ions and sulfate ions had a slight inhibition effect^22^. Černigoj and co-workers (2010) studied the effects of dissolved ozone or ferric ions on the photodegradation of thiacloprid in the presence of TiO_2_ catalysts^23^. They observed that dissolved iron(III) species did not promote the photocatalytic degradation of thiacloprid by TiO_2_. Cruz *et al*.^24^ performed their experiments for the degradation of selected pesticides by bare TiO_2_ and grapheme oxide TiO_2_ under the conditions of ultrapure and natural water^24^. Their results showed that natural water decreased the degradation of four pesticides (diuron, alachlor, isoproturon and atrazine) using bare TiO_2_ as the photocatalyst, however, the degradation of four pesticides was not affected in ultrapure water. They attributed the difference to inorganic and organic species in natural water that inhibited the photocatalytic process. Overview, most of researchers discussed the effects of chemicals on the generation of active free radicals by nanomaterials. Few examples regarding the effects of chemicals on the colloidal stability of nano-TiO_2_ affecting photocatalytic activity are available.

In this work, atrazine was selected as the target pollutant and commercial nano-TiO_2_ particles were employed as the photocatalyst. The effects of Ca^2+^ and/or fulvic acids (FA) on the adsorption and photocatalytic degradation of atrazine were systematically studied. The degradation mechanism was the hypothesis that Ca^2+^ and/or FA could influence the colloidal stability of nano-TiO_2_ particles so as to influence degradation of atrazine. Therefore, the effects of Ca^2+^ and/or FA on colloidal stability of nano-TiO_2_ particles (zeta potential, hydrodynamic diameter (HDD) and sedimentation kinetics) were investigated. The possible relationship between colloidal stability and the photocatalytic properties of nano-TiO_2_ particles in the presence of Ca^2+^ and/or FA was discussed.

## Results and Discussion

### Characterization of commercial nano-TiO_2_

The characteristics of the commercial nano-TiO_2_ with an average particle size of 5–10 nm used in the experiments were shown in Supporting Information (Figs S1–4). As observed in scanning electron microscopy (SEM) image (Fig. S1a), the shape of particles were irregular spheres, and they were aggregated strongly which probably due to thermodynamic stability^25^. Elemental analysis by energy dispersive X-ray (EDX) of the TiO_2_ particles (Fig. S1b) showed that the particles were consisted of Ti (58.76 wt%), O (40.56 wt%) and a small amount of silicon impurity (0.58 wt%). The FT-IR spectrum (Fig. S2) showed that nano-TiO_2_ appeared two absorption peaks at 516.92 and 3437.15 cm^−1^, which corresponded to the vibration of Ti-O-Ti and the adsorption of –OH or H_2_O on nanoparticles respectively. Figure S3 showed that the diffraction peaks at 2θ values of 25.08°, 37.12°, 47.04°, 53.9° and 61.8° of the nanoparticles corresponded to the (101), (004), (200), (211) and (204) planes, respectively. The diffraction peaks were in consistent with the TiO_2_ anatase which were in accordance with the parameters of nano-TiO_2_ supplied by the company. Surface charges of the nano-TiO_2_ particles over the pH range of 1.0–11 were investigated and the point of zero charge of pH (pH_pzc_) was 6.2 (Fig. S4) as with previous reports^26^. TiO_2_ was an amphoteric oxide semiconductor. The nano-TiO_2_ surface was positively charged when pH < 6.2, while at pH > 6.2, the nanoparticles were negatively charged^27^.

1 $${\rm{TiOH}}+{{\rm{H}}}^{+}\to {{{\rm{TiOH}}}_{2}}^{+}({\rm{pH}} < {{\rm{pH}}}_{{\rm{pzc}}})$$

2 $${\rm{TiOH}}+{{\rm{OH}}}^{-}\to {{\rm{TiO}}}^{-}+{{\rm{H}}}_{2}{\rm{O}}\,({\rm{pH}} > {{\rm{pH}}}_{{\rm{pzc}}})$$

These could be important factors for the photocatalysis and colloidal stability of nano-TiO_2_ in different water matrices.

### Effect of Ca^2+^ on the photocatalytic degradation of atrazine

**Figure 1:**
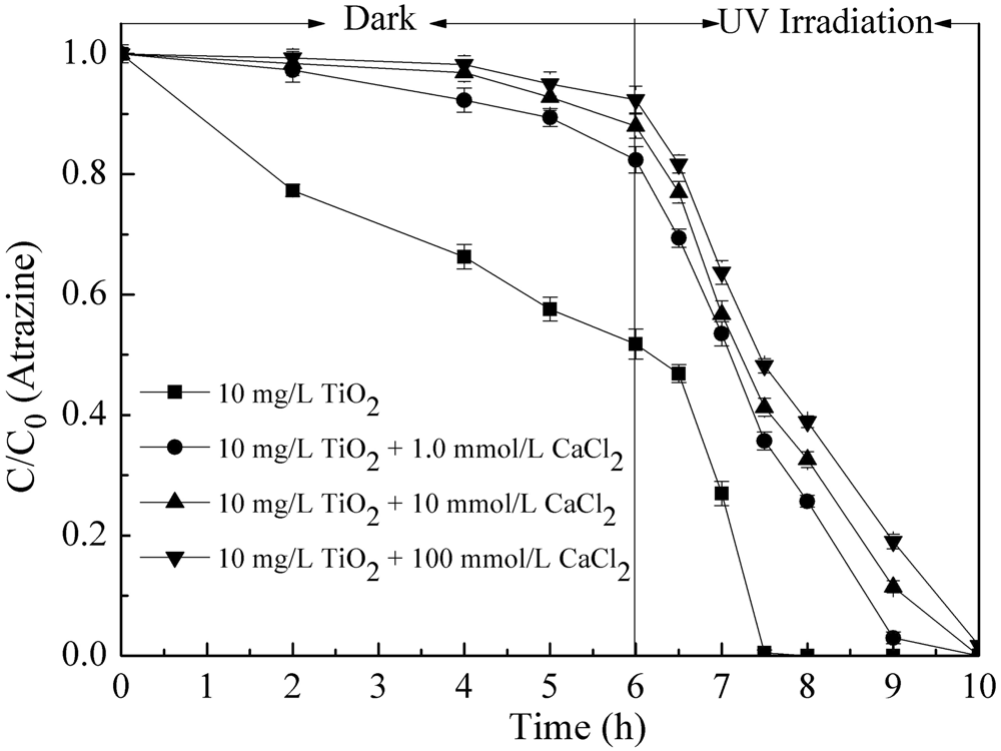
Effect of CaCl_2_ concentration on atrazine removal by nano-TiO_2_. Experimental conditions: TiO_2_ concentration 10 mg/L, atrazine concentration 1.0 mg/L, pH 7.0.

The effect of Ca^2+^ on the removal of atrazine by nano-TiO_2_ was evaluated in suspensions with 10 mg/L of nano-TiO_2_, 1.0 mg/L of atrazine and different concentrations of CaCl_2_ at pH 7.0. Results were presented in Fig. 1. In the first six hours of darkness, adsorption on nano-TiO_2_ surface was the primary mechanism for the removal of atrazine, and then followed by photocatalytic degradation in the remaining four hours of UV irradiation.

During the dark period, adsorption of atrazine onto the nano-TiO_2_ surface decreased significantly with the addition of Ca^2+^. Without Ca^2+^, the removal efficiency of atrazine by nano-TiO_2_ was 49.2% after six hours. The addition of 1.0 mmol/L CaCl_2_ resulted in a decrease in adsorption to 17.6%, and the removal efficiency decreased to 7.6% when the CaCl_2_ concentration was increased to 100 mmol/L. During UV irradiation, the photocatalytic degradation efficiency of atrazine also decreased with the addition of Ca^2+^. With no addition of CaCl_2_, the photodegradation rate was approximately 0.46 C/C_0_/hr (from hour 6.5 to 7.5). However, the increase of CaCl_2_ from 1.0 to 100 mmol/L resulted in a lower rate of 0.35 to 0.32 C/C_0_/hr during the same time period. Atrazine was completely degraded after 2.0 hours of UV irradiation with the absence of CaCl_2_. Nevertheless, when the concentration of CaCl_2_ was 100 mmol/L, there was still 38.9% of atrazine left in the solution after 2.0 hours of photocatalytic process, and it wasn’t completely degraded till the experiment finished. Similar results were reported by other investigators for other organic pollutants. Dionysiou and co-workers (2000) studied the influences of KNO_3_ and H_2_O_2_ on the removal of 4-chlorobenzoin (4-CBA) by TiO_2_ powders^28^. They found that the removal efficiency of 4-CBA by TiO_2_ decreased with the increase of KNO_3_ concentration at both adsorption and photocatalytic degradation processes. Maybe due to the high TiO_2_ loading, the complete degradation of 4-CBA was achieved at 3.0 h which was faster than that in our study.

**Figure 2:**
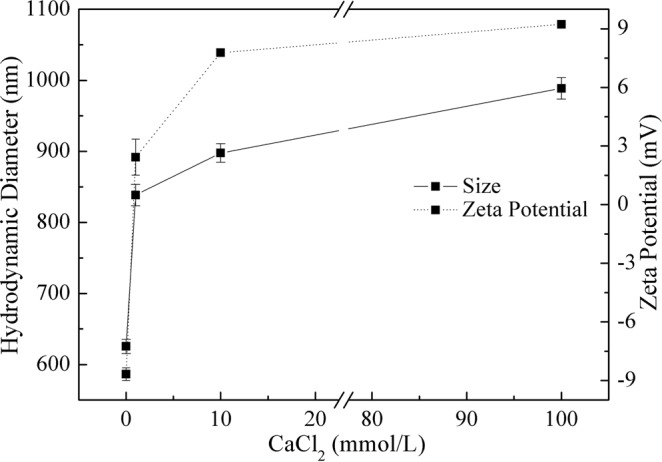
Effect of CaCl_2_ concentration on HDD and zeta potential of nano-TiO_2_. Experimental conditions: TiO_2_ concentration 10 mg/L, pH 7.0.

In order to explained the mechanism of atrazine removal by nano-TiO_2_ with the increase of Ca^2+^ concentration, the colloidal stability (zeta potential and HDD) of nano-TiO_2_ suspensions (pH 7.0) in different concentrations of CaCl_2_ was studied systematically. The results were shown in Fig. 2. With the addition of CaCl_2_ concentration, the HDD of nano-TiO_2_ particles increased and the zeta potential changed from negative to positive values but still near zero, which meant the aggregation occurred between the nanoparticles. These changes could have reduced the actual contact surface area and active adsorption sites of nano-TiO_2_ thus decreasing the adsorption capacity of nanoparticles for organic pollutants^29^. This suggested that the absorption and photodegradation mechanism of atrazine behaved much differently in distilled water and simulating natural waters.

**Figure 3:**
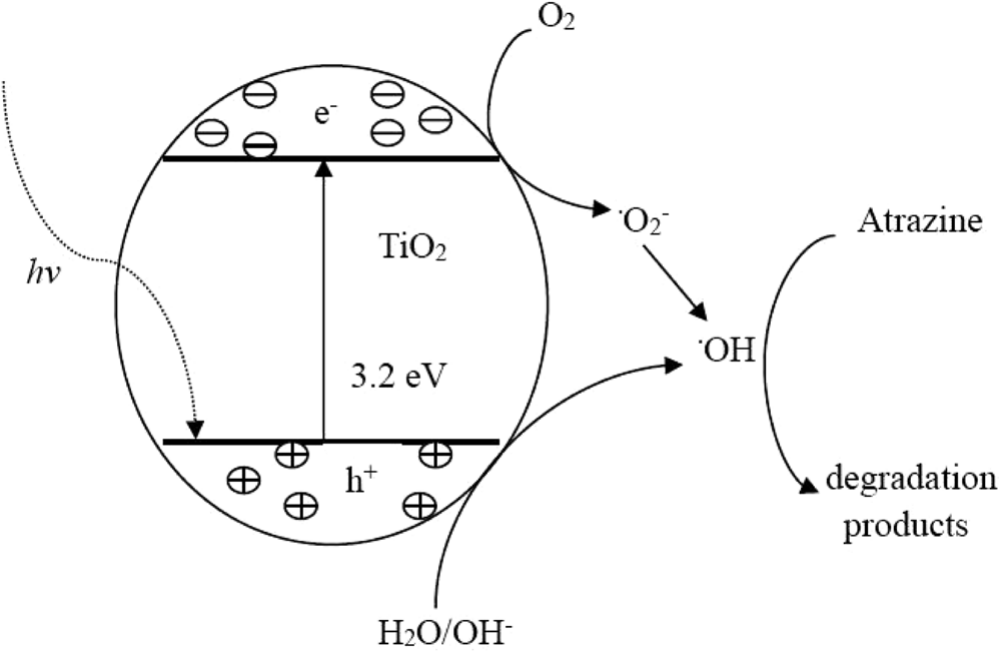
Photocatalytic degradation pathway of atrazine by nano-TiO_2_.

According to the report by Chen and Liu^27^, the photocatalytic mechanism in the presence of TiO_2_ could be described by Fig. 3 and the equations as follows:

3 $${{\rm{TiO}}}_{2}+hv\to {{\rm{TiO}}}_{2}+{{\rm{e}}}^{-}+{{\rm{h}}}^{+}$$

4 $${{\rm{O}}}_{2}+{{\rm{e}}}^{-}\to \,{}^{\cdot }{\rm{O}}_{2}^{-}$$

5 $${{\rm{H}}}_{2}{\rm{O}}+{{\rm{h}}}^{+}\to {}^{\cdot }{\rm{OH}}^{-}+{{\rm{H}}}^{+}$$

6 $${{\rm{OH}}}^{-}+{{\rm{h}}}^{+}\to {}^{\cdot }{\rm{OH}}$$

7 $${\rm{atrazine}}+{}^{\cdot }{\rm{OH}}\to {\rm{degradation}}\,{\rm{products}}$$

From the above equations and Fig. 3, the hydroxyl radical (OH) was the main reactant produced by UV irradiation on the surface of TiO_2_ for atrazine photocatalytic degradation. When adding Ca^2+^, the aggregation of nanoparticles increased the contact thereby decreasing the exposed surface area and the generation of hydroxyl radicals. These were resulted in a reduction of photocatalytic degradation efficiency^30^. From Figs 1 and 2, the mechanism of atrazine removal by nano-TiO_2_ at the CaCl_2_ solution could be well explained by the colloidal stability of nano-TiO_2_. However, it should be noted that the decrease trend of photocatalytic degradation efficiency was not high as expected in Ca^2+^ concentration.

### Effects of Ca^2+^ and FA on the photocatalytic degradation of atrazine

**Figure 4:**
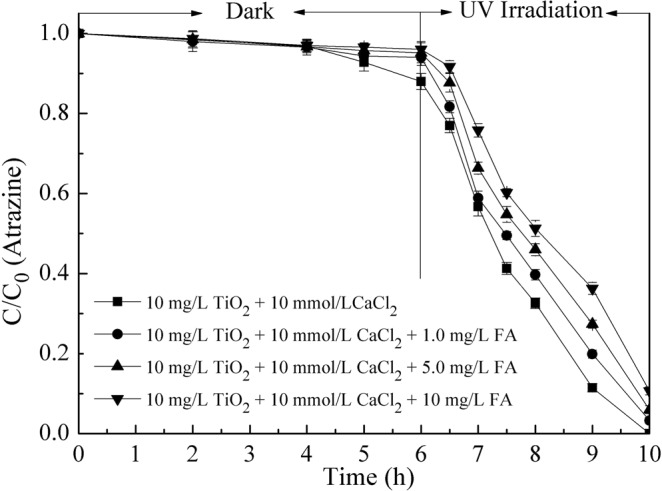
Effects of Ca^2+^ and FA on atrazine removal by nano-TiO_2_. Experimental conditions: TiO_2_ concentration 10 mg/L, atrazine concentration 1.0 mg/L, CaCl_2_ concentration 10 mmol/L, pH 7.0.

To determine the combined effects of Ca^2+^ and FA on the removal of atrazine by nano-TiO_2_, experiments were performed in the suspensions with 10 mg/L of nano-TiO_2_, 1.0 mg/L of atrazine, 10 mmol/L of CaCl_2_ and increasing concentrations of FA at pH 7.0. The results were shown in Fig. 4. During the dark period of the experiments, the adsorption of atrazine onto the nano-TiO_2_ decreased slightly with increasing FA concentration from 1.0 to 10 mg/L. During UV irradiation, complete photocatalytic degradation of atrazine by nano-TiO_2_ was only obtained after 10 hours in the absence of FA, and over 90% of atrazine was degraded in the solutions containing FA. In addition, the higher amount of FA added, more residual atrazine was left in the solution, which suggested that FA inhibited the removal capacity of nano-TiO_2_. The results obtained in this study were agreed with the research by Wang *et al*. (1999) which investigated the effects of pH, inorganic ions and humic acids on the photocatalytic degradation of 2-chlorobiphenyl (2-CB) by TiO_2_^31^. However, in their study, the decrease of 2-CB degradation with the increase of humic acids concentration was ascribed to the competition between humic acid and 2-CB.

**Figure 5:**
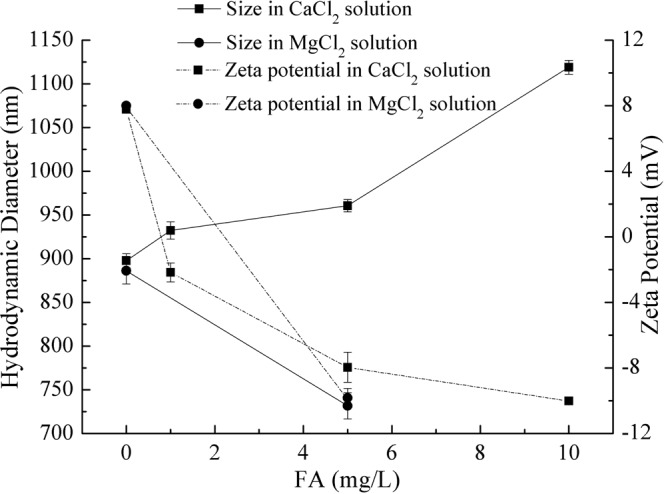
Effect of FA concentration on HDD and zeta potential of nano-TiO_2_. Experimental conditions: TiO_2_ concentration 10 mg/L, CaCl_2_ and MgCl_2_ concentration all 10 mmol/L, pH 7.0.

The results of atrazine degradation in the presence of Ca^2+^ and FA could be attributed to the colloidal stability of nanoparticles suspensions and the competition for hydroxyl radicals by FA^32^. The presence of Ca^2+^ and NOM, such as FA, could form ion bridge effects resulting in intensified aggregation of nanoparticles^33^. The aggregation could reduce the active adsorption sites of nano-TiO_2_, thereby decreasing the adsorption capacity of nanoparticles. The HDD and zeta potential of nano-TiO_2_ suspensions in the presence of Ca^2+^ and FA were investigated and the results were presented in Fig. 5. When the solution containing 10 mmol/L CaCl_2_, the HDD of nano-TiO_2_ increased with the increase of FA concentration. Moreover, FA had a high adsorption capacity and could compete for active adsorptive sites of nano-TiO_2_^34,35^. From Fig. 4, the adsorption efficiency of atrazine decreased with the addition of FA in the first six hours. The results could well validate the analysis acquired by the above-mentioned research. During UV irradiation, on the one hand, the aggregation of nanoparticles decreased the generation of hydroxyl radical because of the recombination of generated holes with electrons from adjacent nanoparticles^30^. On the other hand, FA as one kind of organic matter could quench hydroxyl radical generated by nano-TiO_2_ under the UV irradiation^31^. Moreover, the photocatalytic degradation efficiency of atrazine by nano-TiO_2_ decreased with the increase of FA, which could also be related with the quenching effect of FA.

**Figure 6:**
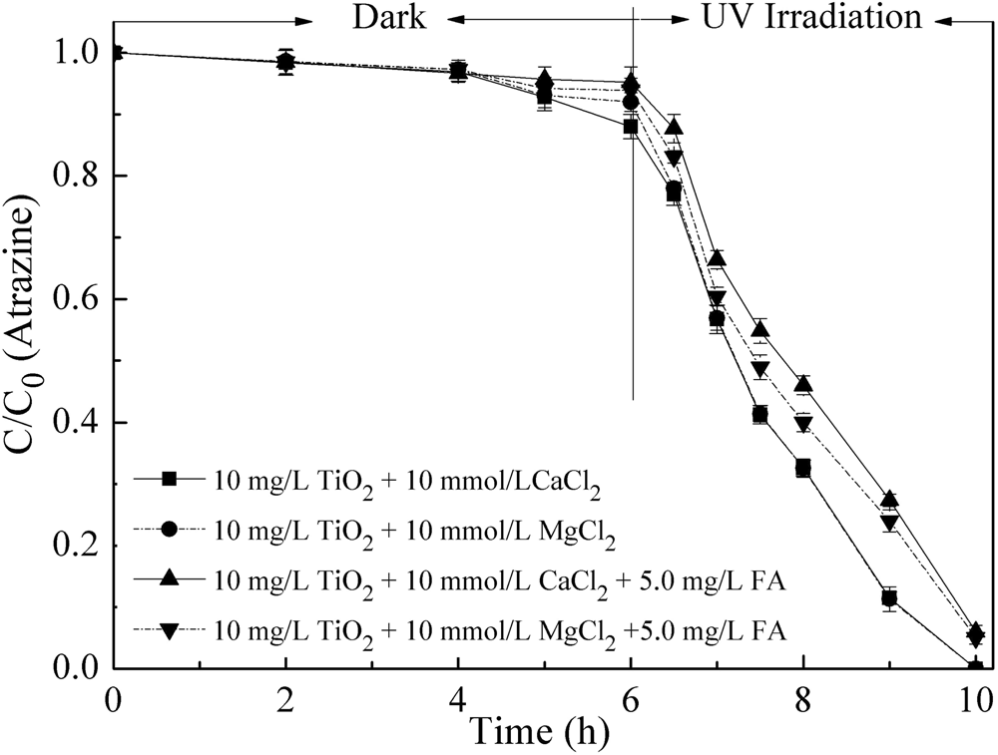
Effects of Ca^2+^, Mg^2+^ and FA on atrazine removal by nano-TiO_2_. Experimental conditions: TiO_2_ concentration 10 mg/L, atrazine concentration 1.0 mg/L, pH 7.0.

In order to well demonstrate the combined effect of Ca-FA, the effects of Mg^2+^ and FA on the colloidal stability and photocatalytic activity of nano-TiO_2_ were also studied. The experiments were performed as above and the results were shown in Figs 5 and 6. Comparing with the addition of 10 mmol/L CaCl_2_, the adsorption and photocatalytic degradation efficiencies of atrazine by nano-TiO_2_ were almost the same in the presence of 10 mmol/L MgCl_2_ (Fig. 6). And from Fig. 5, it could be seen that the nanoparticles were also similar in size. However, when in metal ion-FA coexistent system, the influence on the property of nano-TiO_2_ was different. When the solution containing MgCl_2_-FA, the HDD of nano-TiO_2_ was smaller and the degradation efficiencies of atrazine by nanoparticles were higher than in the CaCl_2_-FA solution. The reason could be that the ion bridge effects couldn’t form in the Mg-FA system^36^. So in this condition, the nanoparticles were dispersed because of the presence of FA. The results could well reveal the effects of Ca^2+^ and FA on the photocatalytic activity of nano-TiO_2_.

### Aggregation of nano-TiO_2_ by the analyses of SEM

**Figure 7:**
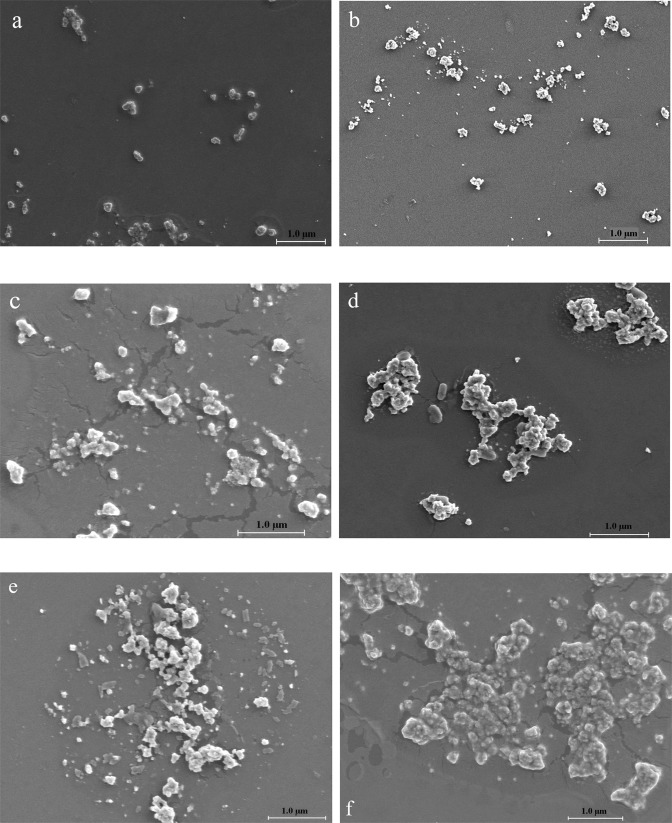
The SEM images of 10 mg/L nano-TiO_2_. (**a**) 1.0 mmol/L CaCl_2_, (**b**) 10 mmol/L CaCl_2_, (**c**) 100 mmol/L CaCl_2_, (**d**) 10 mmol/L CaCl_2_ + 1.0 mg/L FA, (**e**) 10 mmol/L CaCl_2_ + 5.0 mg/L FA, (**f**) 10 mmol/L CaCl_2_ + 10 mg/L FA.

To be able to visually study the aggregation of nano-TiO_2_ in solutions containing Ca^2+^ and/or FA, six samples were selected and measured by SEM. The six samples were that 10 mg/L nano-TiO_2_ particles were mixed into solutions (pH 7.0) containing 1.0 mmol/L CaCl_2_, 10 mmol/L CaCl_2_, 100 mmol/L CaCl_2_, 10 mmol/L CaCl_2_ and 1.0 mg/L FA, 10 mmol/L CaCl_2_ and 5.0 mg/L FA, 10 mmol/L CaCl_2_ and 10 mg/L FA, respectively. The SEM images were shown in Fig. 7.

From Fig. 7, it could be seen that the size of nano-TiO_2_ was smallest in the solution cotaining 1.0 mmol/L CaCl_2_. Increasing CaCl_2_ concentration, the degree of nano-TiO_2_ aggregation was increased (Fig. 7a–c). When FA was added to the 10 mmol/L CaCl_2_ solution, nano-TiO_2_ particles were gathered together and the size of nanoparticles were increased sharply (Fig. 7d). Even 1.0 mg/L FA added, the size of nanoparticles was bigger than that in the solution containing CaCl_2_. With the increase of FA concentration, the aggregation phenomena was getting serious (Fig. 7d–f). The results of SEM images were in accordance with the HDD of nanoparticles measured by the Nano ZS90 Malvern Zetasizer (Figs 2 and 5).

### Effect of nano-TiO_2_ sedimentation on the photocatalytic degradation

**Figure 8:**
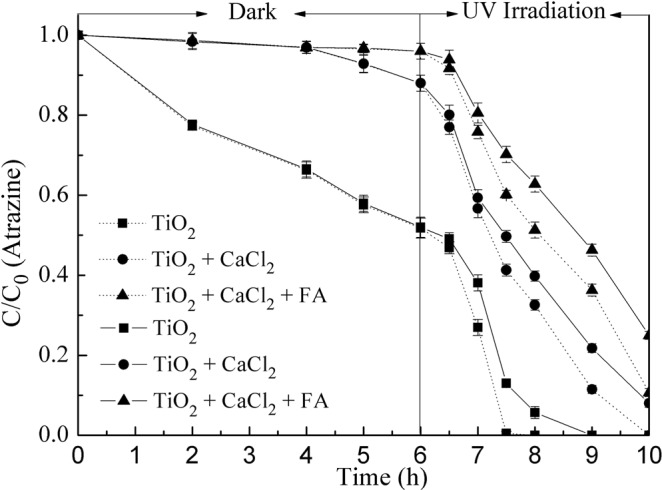
Effects of CaCl_2_ and FA on atrazine removal by nano-TiO_2_. Dashed lines (…) mean suspension effect, solid lines (—) mean sedimentation effect. Experimental conditions: TiO_2_ concentration 10 mg/L, atrazine concentration 1.0 mg/L, CaCl_2_ concentration 10 mmol/L, FA concentration 10 mg/L, pH 7.0.

In order to evaluate the effect of nano-TiO_2_ sedimentation on the photocatalytic degradation of atrazine in the presence of Ca^2+^ and FA, experiments were conducted in suspensions with 10 mg/L TiO_2_, 1.0 mg/L atrazine, 10 mmol/L CaCl_2_ and 10 mg/L FA at pH 7.0. From Fig. 8, it was found that the photocatalytic degradation efficiency of atrazine was lower under the sedimentation conditions compared with the suspended situation under all conditions. When only 10 mg/L of TiO_2_ was existed, full photocatalytic degradation of atrazine occurred after 1.5 hours of UV irradiation under the suspended situation, while complete degradation was delayed by 1.5 hours under the condition of sedimentation. With the addition of 10 mmol/L CaCl_2_ and after 4.0 hours of UV irradiation, 8.0% of atrazine remained in the solution under sedimentation condition, while it was degraded completely under suspension conditions. Similar results were observed with FA addition. The sedimentation caused an adverse impact on the photocatalytic performance of nano-TiO_2_.

**Figure 9:**
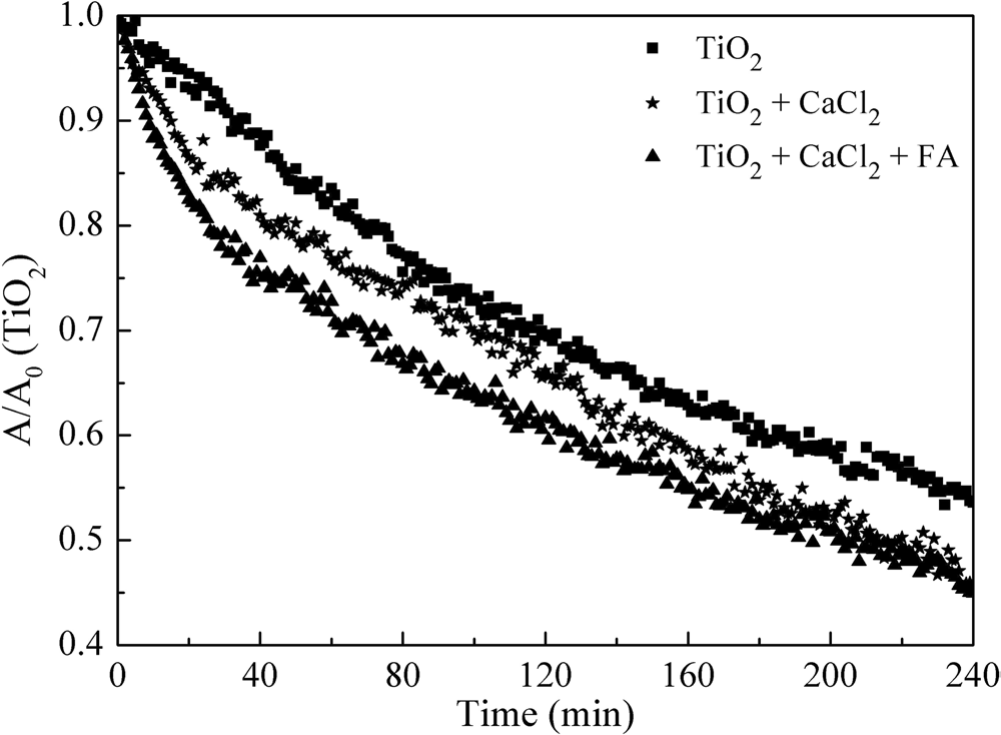
Sedimentation of nano-TiO_2_. Experimental conditions: TiO_2_ concentration 10 mg/L, CaCl_2_ concentration 10 mmol/L, FA concentration 10 mg/L, pH 7.0.

The reduction in photocatalytic degradation efficiency of atrazine by nano-TiO_2_ under sedimentation was explained by the decrease in the number of nano-TiO_2_ particles (Fig. 9). Under sedimentation condition, the smaller number of nano-TiO_2_ particles resulted in less surface area for UV exposure, and the output of hydroxyl radical was decreased for organic pollutant oxidation^37^. Fast sedimentation could decrease the contact time between nanoparticles and target pollutants, which could quench the hydroxyl radical produced by nano-TiO_2_^38^. This decreased the photocatalytic performance of nano-TiO_2_. Sedimentation was fastest in the CaCl_2_ and FA coexistent system and followed by CaCl_2_ alone, supporting the contention that the addition of Ca^2+^ and Ca^2+^-FA badly affected the colloid stability and catalytic activity of nano-TiO_2_ particles.

## Conclusion

In this study, attrazine degradation efficiency and colloidal stability of nano-TiO_2_ were investigated systematically. The adsorption and photocatalytic degradation of atrazine by nano-TiO_2_ were negatively affected by the addition of Ca^2+^ and fulvic acids (FA) in aqueous solutions. The results suggested that the removal of atrazine by nano-TiO_2_ was controlled by colloidal stability and adsorption interferences in the presence of Ca^2+^ and/or FA. The addition of Ca^2+^ could cause aggregation of nanoparticles by compressing the electric double layer, while FA could interfere by competitive adsorption. In photocatalytic degradation, the increase of particle size decreased the generation of hydroxyl radical. Besides, FA could quenche the hydroxyl radicals, thereby reducing the degradation efficiency of atrazine. Under sedimentation conditions, the number of nano-TiO_2_ particles decreased in all solutions. Due to smaller available surface area, the photocatalytic degradation of atrazine decreased. During the sedimentation, the number of nano-TiO_2_ particles remaining in solutions containing Ca^2+^ and Ca^2+^-FA was less than the control, which demonstrated the negative effect of Ca^2+^ and FA on the colloidal stability and catalytic activity of nano-TiO_2_ particles.

## Methods

### Preparation of reagents

Commercial TiO_2_ (anatase) nanoparticles (nano-TiO_2_) were provided by Aladdin Chemistry Co. Ltd. The average particle size was 5–10 nm and the content of TiO_2_ was over 99.8% as reported by the company. Atrazine of analytical grade was purchased from Shanghai Yuanye Bio-technology Co., Ltd, China, and stored at 4 °C before the experiment. FA with a molecular weight of 308.24 g/mol was obtained from the Shanghai Luzong Chemical Reagent Co., Ltd without additional purification. Other chemical reagents employed in this study, including CaCl_2_, MgCl_2_, NaOH and HCl, were all of reagent grade and obtained from Damao Chemical Reagent Co, Tianjin, China.

A nano-TiO_2_ stock solution (50 mg/L) was prepared immediately before use with ultrapure water (Barnstead D11911), and sonicated at 25 °C for 30 min with the ultrasonic power of 100 W and frequency of 40 kHz. Atrazine was dissolved in ultrapure water to obtain the 10 mg/L stock solution and stored at 4 °C without light. FA stock solution with a concentration of 1000 mg/L was prepared by dissolving FA in ultrapure water and stored at 4 °C before the experiment. A stock solution of CaCl_2_ (1.0 mol/L) used as Ca^2+^ was prepared in the same manner. The 1.0 mol/L stock solution of MgCl_2_ was prepared as above. The chloride ion was chosen as anion in the experiments due to its little influence on the degradation and colloidal stability of nano-TiO_2_^39^.

When performing a given experiment, the concentrations of all reagents used were prepared by diluting the stock solution. All containers used in the study were washed and dried carefully to prevent dust interference.

### Atrazine degradation by nano-TiO_2_ in the presence of Ca^2+^ and/or FA

#### Photocatalytic reactor

In this study, a small self-made ultraviolet photocatalytic reactor which was also used in our other experiment was employed for atrazine removal^40^. Photocatalytic experiments were performed in a cylindrical Pyrex glass cylinder (diameter 9.0 cm, height 12 cm) containing a 100 mL aqueous sample under a 15 W tube-like ultraviolet lamp (GPH843T5VH, Longpro Co., Ltd, Guangzhou, China). The solutions were stirred by a 85–2 digital magnetic stirrer (Changzhou Guoyu instrument manufacturing co., LTD, Jiangsu, China) at 250 rpm. The distance between ultraviolet lamp and solution surface was 25 cm in order to maintain a fixed intensity of light. A lightproof casing was used for avoiding the contact of photocatalytic simulator with outside.

#### Photocatalytic experiments

The photocatalytic experiments containing nano-TiO_2_ particles and atrazine were performed with CaCl_2_, MgCl_2_ and/or FA. The experiments were carried out at pH 7.0 and a temperature of 30 °C. Each experiment lasted for 10 hours and was divided into two parts: 6.0 hours of darkness (0–6 h) and then followed by 4.0 hours of UV illumination (6–10 h)^28^. In the experiments, the concentrations of nano-TiO_2_ and atrazine were 10 and 1.0 mg/L, respectively, and the total volume of the mixture was 100 mL. In order to obtain a well dispersed solution, the experimental timing began after stirring for 10 min at 250 rpm. 1.0 mL samples were taken out from the reactor at set times and filtered with a 0.45 μm nylon syringe filter. The atrazine concentration of the sample was measured. Sampling times were 0, 2.0, 4.0, 5.0, 6.0, 6.5, 7.0, 7.5, 8.0, 9.0, 10 h.

To study the effects of Ca^2+^ concentrations on the atrazine degradation by nano-TiO_2_, proper volumes of stock CaCl_2_ solution and ultrapure water were added to the mixture at the 4^th^ hour. To reduce experimental error, the additional total volume of stock solution and ultrapure water was 1.0 mL in all experiments. When CaCl_2_ was added to the solutions, NaOH or HNO_3_ was added quickly to readjust the pH to 7.0. To reduce the additional volume of acid or base, special care was taken to add as little HNO_3_ or NaOH as possible. To evaluate the effects of Ca^2+^ and FA on the atrazine degradation, 10 mmol/L CaCl_2_ and FA (1.0, 5.0 10 mg/L) were added at the very beginning and the 4^th^ hour respectively. The other experiments were performed as above. The effects of Mg^2+^ and FA on the atrazine degradation by nano-TiO_2_ were conducted as above.

In order to study the effect of TiO_2_ sedimentation on the atrazine photocatalytic degradation at the concentrations of 10 mmol/L CaCl_2_ and/or 10 mg/L FA, the experiment of atrazine degradation by nano-TiO_2_ was the same as above except that stirring was stopped during the 4.0 hours of UV illumination.

All photocatalytic experiments were conducted in duplicate and the average atrazine concentration was used to analyze the result.

### Colloidal stability of nano-TiO_2_ suspension

Colloidal stability of nano-TiO_2_ particles were studied by examining the zeta potential and HDD of nano-TiO_2_ particle suspensions in the presence of Ca^2+^ and/or FA at pH 7.0. For all colloidal stability experiments, the concentrations of nano-TiO_2_ particles were 10 mg/L. The suspensions containing CaCl_2_ (1.0, 10, 100 mmol/L) and/or FA (1.0, 5.0, 10 mg/L) were prepared by adding appropriate volumes of the stock solutions and stirring at 250 rpm, 25 °C for 30 min. The zeta potential and HDD of the nano-TiO_2_ suspensions were measured immediately after stirring. All aggregation experiments were carried out in duplicate and the average values were used for analysis.

To evaluate the sedimentation kinetics of nano-TiO_2_ particles in the present of Ca^2+^ and/or FA, the mixture containing 10 mg/L nano-TiO_2_, 10 mmol/L CaCl_2_ and/or 10 mg/L FA was stirred at 250 rpm, 25 °C for 30 min. Then the absorbance (A) of nano-TiO_2_ was measured in drive-time mode for 4.0 hours^41^. Control experiments containing 10 mg/L of nano-TiO_2_ were carried out in parallel.

### Photocatalyst characterization

The surface morphology and sample dimensions of the commercial nano-TiO_2_ were determined by SEM (FEI QuANTA 200, USA)^42^. Quantitative detection and localization of elements in the photocatalyst were measured using an energy dispersive X-ray (EDX). The FT-IR spectrum was measured by a Fourier transform infrared spectrometer (Infinity-1, Shimadzu, Japan) in the range of 400–4000 cm^−1^. A Bruker AXS D8 advance diffractometer with Cu radiation under 40 kV and 250 mA was employed for measuring the X-ray diffraction (XRD) patterns of nanoparticles. The pH_pzc_ of the nano-TiO_2_ particles was measured by a Nano ZS90 Malvern Zetasizer (Malvern Instrument, Worcestershire, UK)^43^.

In order to measure the morphology of nano-TiO_2_ in solutions containing Ca^2+^ and/or FA, the mixed solution was stirred for 30 min, and taken out a drop of sample to a clean silicon wafer (1.0 cm × 1.0 cm). Then the sample was dried for 24 h by a vacuum freeze dryer (WLFD-1–50, Beijing Bairui Weilai Analysis instrument co., LTD). After dried, the sample was sprayed gold for 45 s and was evaluated by the SEM (FEI QuANTA 200, USA).

### Analytical methods

High performance liquid chromatography (HPLC) (Agilent 1100 Series with quaternary pump) with a C18 column UV detector (5 um, 4.6 × 150 mm) was employed to analyze the concentration of atrazine (C)^44^. 20 μL samples were injected into the instrument and monitored at 230 nm for 7.0 min. The mobile phase was kept constant at 30% HPLC grade water, 60% HPLC grade methyl alcohol and 10% HPLC grade acetonitrile. The flow rate was 1.0 mL/min and the measuring temperature was 40 °C.

The Nano ZS90 Malvern Zetasizer was employed to measure the zeta potential and HDD. The zeta potential was determined from the electrophoretic mobility by the Smoluchowski model, and HDD was obtained from the diffusion coefficient by the Stokes-Einstein equation^33,45^. For each sample, the zeta potential value was obtained from the average of 30 measurements and HDD was measured once. Before measuring, the Malvern Zetasizer was performed at 25 °C for 1.0 min to equilibrium^33^. A new disposable folded capillary cell and polystyrene cuvette were used to measure the zeta potential and HDD for each sample, respectively.

The absorbance of nano-TiO_2_ was measured by an UV-Vis spectrophotometer (UV-2550, Shimadzu, Japan) at 343 nm for the sedimentation kinetics experiments. The absorbance values were obtained every minute for 4.0 hours for each sample. The temperature was kept at 25 °C during the experiment.

### Ethical statement

This article does not contain any studies with human participants or animals performed by any of the authors.

## Supplementary information

Supplementary Information
