# Supplementary material for: Effects of Ca2+ and fulvic acids on atrazine degradation by nano-TiO2: Performances and mechanisms
Source: Sci Rep. 2019 Jun 20;9:8880. doi: 10.1038/s41598-019-45086-2 (PMC6586927; doi:10.1038/s41598-019-45086-2)
Supplement: Supplementary file 1 — Supplementary Information [file 41598_2019_45086_MOESM1_ESM.doc]

**Supplementary Information**

**Effects of Ca^2+^ and fulvic acids on atrazine degradation by nano-TiO_2_: Performances and mechanisms**

Saiwu Sun^3,4^, Huijun He^1,3,*^, Chunping Yang^2,3,*^, Yan Cheng^1^, Yongpan Liu^1^

1. College of Environmental Science and Engineering, Guilin University of Technology and Guangxi Key Laboratory of Theory & Technology for Environmental Pollution Control (Guilin University of Technology), Guilin, Guangxi 541004, China;

2. Guangdong Provincial Key Laboratory of Petrochemical Pollution Process and Control, School of Environmental Science and Engineering, Guangdong University of Petrochemical Technology, Maoming, Guangdong 525000, China;

3. College of Environmental Science and Engineering, Hunan University and Key Laboratory of Environmental Biology and Pollution Control (Hunan University), Ministry of Education, Changsha, Hunan 410082, China;

4. Hunan Dalu Technology Co., Ltd, 559 Yunxi Road, Changsha, Hunan 410036, China.

^*^ Corresponding authors. Email: [hehuiijun@hnu.edu.cn](mailto:hehuiijun@hnu.edu.cn) (H.J. He), [yangc@hnu.edu.cn](mailto:yangc@hnu.edu.cn;) (C.P. Yang)

# These authors contribute equally to this paper.

**Photocatalyst characterization**

The surface morphology and sample dimensions of the commercial nano-TiO_2_ were determined by SEM (FEI QuANTA 200, USA). Quantitative detection and localization of elements in the photocatalyst were measured using an energy dispersive X-ray (EDX). The FT-IR spectrum was measured by the Fourier transform infrared spectrometer (Infinity-1, Shimadzu, Japan) in the range of 400-4000 cm^−1^. A Bruker AXS D8 advance diffractometer with Cu radiation under 40 kV and 250 mA was employed for measuring the X-ray diffraction (XRD) patterns of nanoparticles. The pH_pzc_ of the nano-TiO_2_ particles was measured by a Nano ZS90 Malvern Zetasizer (Malvern Instrument, Worcestershire, UK).


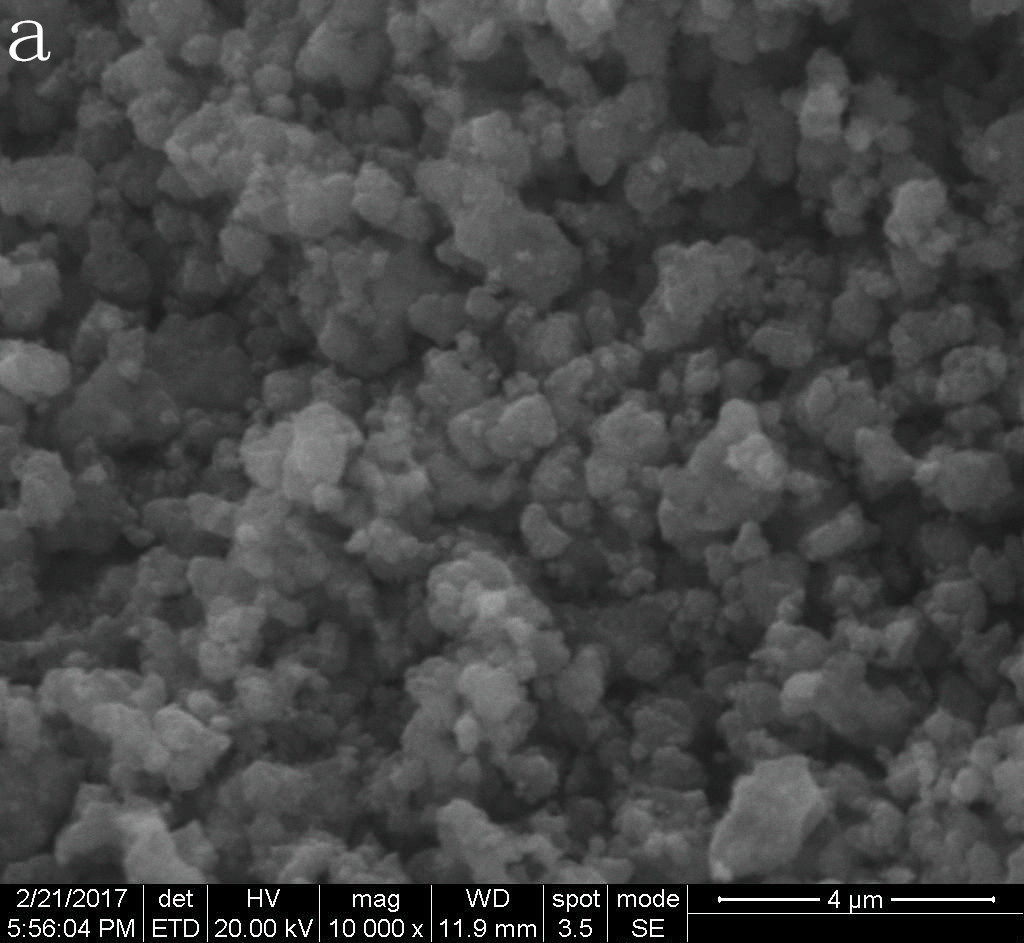

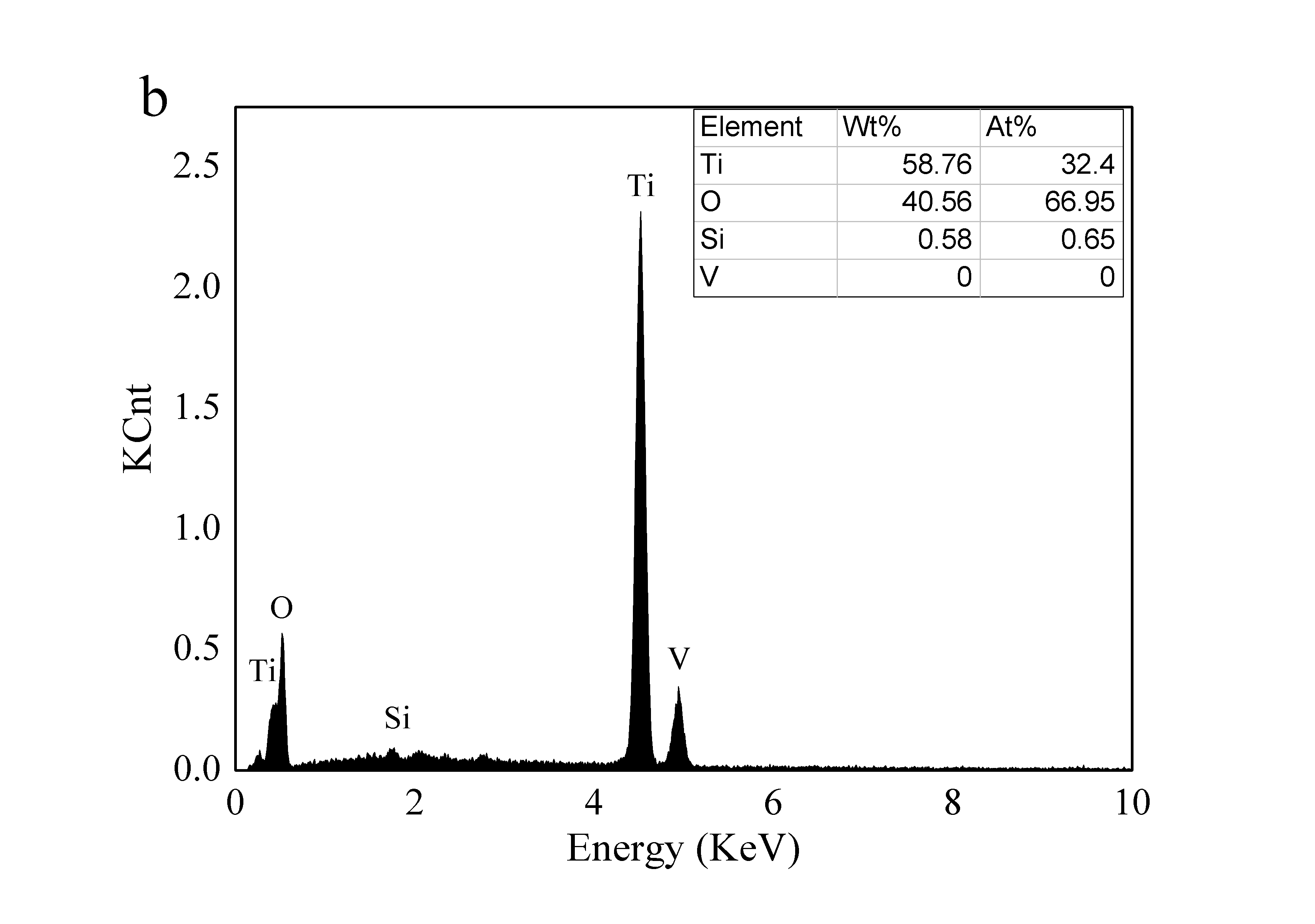


**Fig. S1.** The SEM image (a) and EDX analysis of commercial nano-TiO_2_.


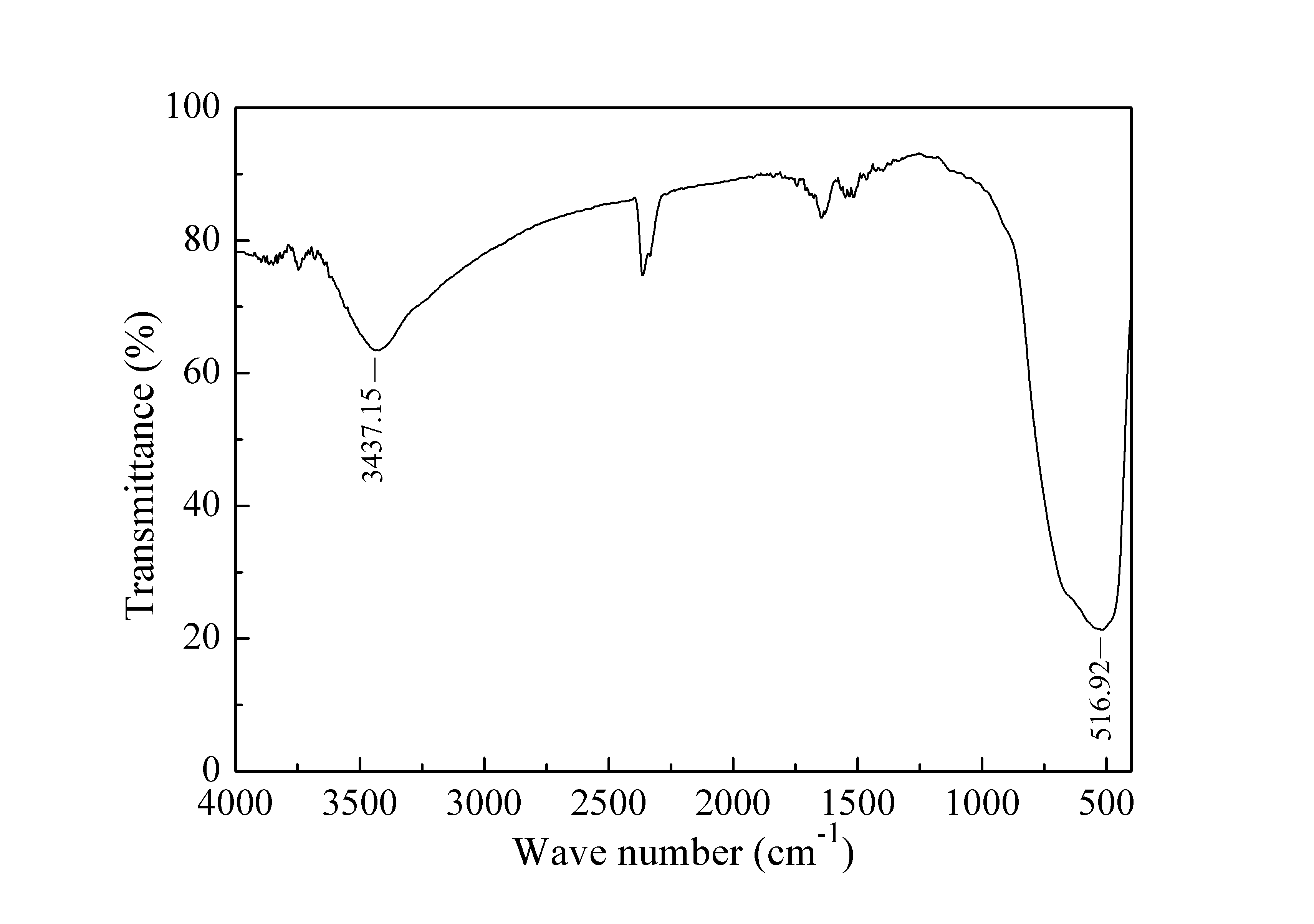


**Fig. S2.** FT-IR spectrum of commercial nano-TiO_2_.


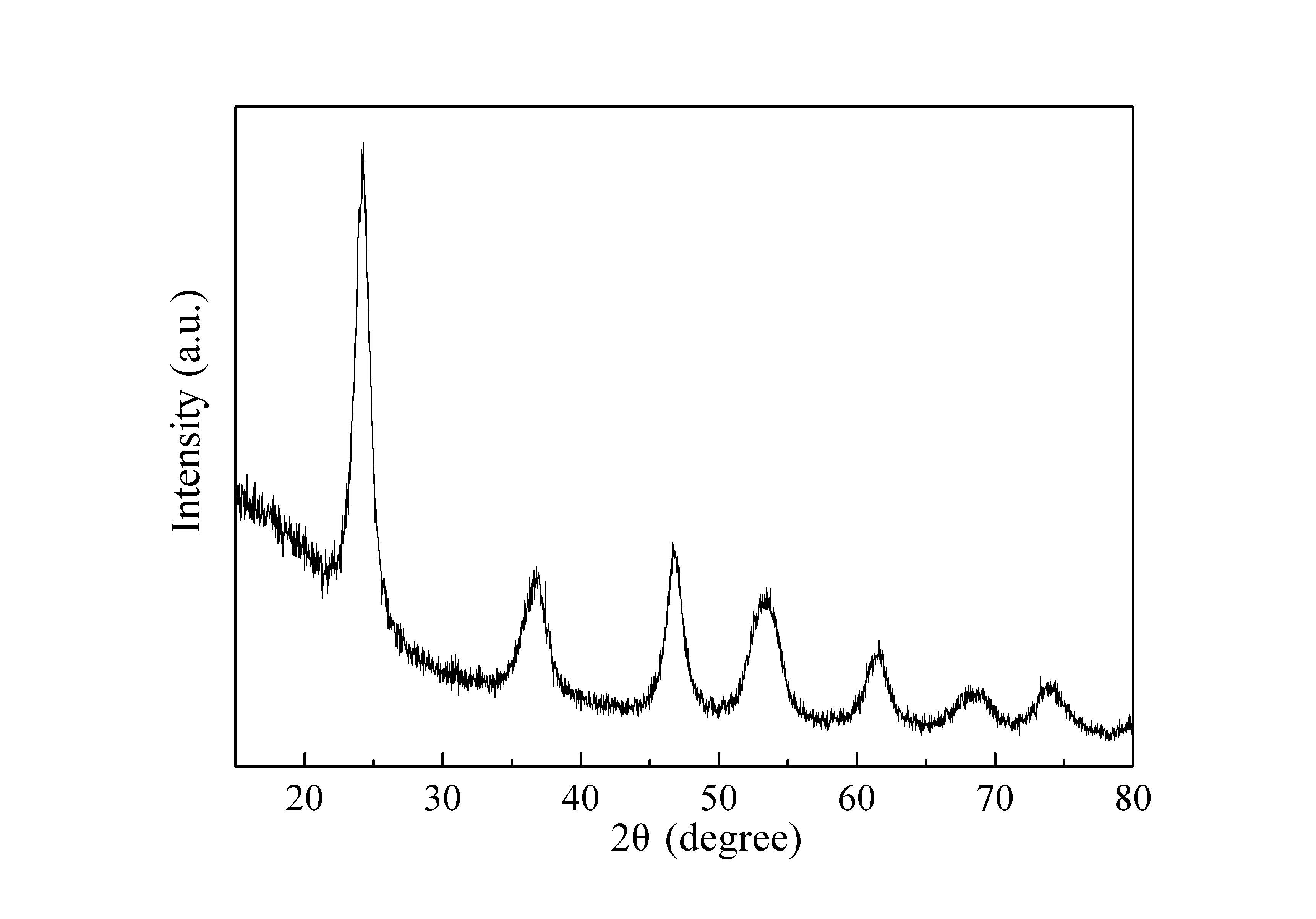


**Fig. S3.** The XRD patterns of commercial nano-TiO_2_.


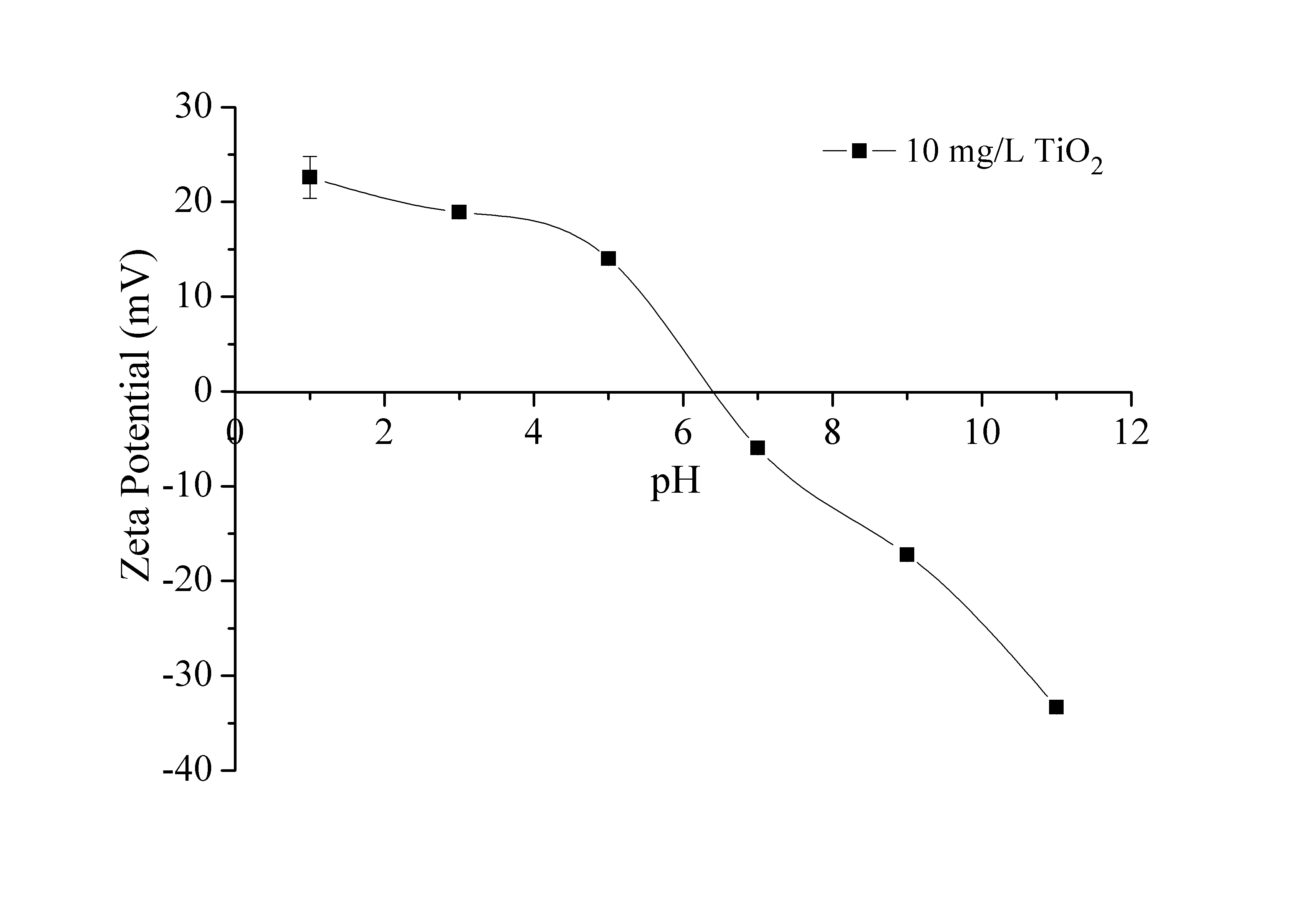


**Fig. S4.** Relationship of Zeta potential between commercial nano-TiO_2_ and pH value.
